# Alteration of ocular surface mucins in MUC5AC-DTA transgenic mice

**Published:** 2009-01-16

**Authors:** I-Jong Wang, Chong-Jen Yu, Fung-Rong Hu

**Affiliations:** 1 Department of Ophthalmology, National Taiwan University Hospital, Taipei, Taiwan; 2 Department of Internal Medicine, National Taiwan University Hospital, Taipei, Taiwan

## Abstract

**Purpose:**

To investigate the compensation of secretory mucins with membranous mucins in mice with goblet cell deficiency.

**Methods:**

A transgenic mouse model in which conjunctival goblet cells were targeted was generated, and the expression of mucins was evaluated through the toxicity of diphtheria toxin A driven by a human mucin, MUC5AC, promoter. Immunohistochemical staining, in situ hybridization, electronic microscopy, and quantitative reverse transcription polymerase chain reaction (RT–PCR) were used to characterize their phenotypes.

**Results:**

The external appearance of the ocular surface was normal, and no corneal pathology was found. The quantity of MUC5AC and the number of conjunctival goblet cells decreased in this mouse as expected. However, the membranous mucin, MUC4, compensates the decrease of MUC5AC in terms of the results of immunohistochemical staining, in situ hybridization, electronic microscopy, and quantitative RT–PCR.

**Conclusions:**

The membranous mucin, MUC4, can compensate for the deficiency of the secretory mucin, MUC5AC, in goblet cell deficient mice. This compensation may explain why the symptoms of mucus threads can be found in some goblet deficiency diseases, and it may provide an alternative defensive mechanism in goblet cell deficiency.

## Introduction

The tear film is composed of an outer lipid layer and an inner aqueous phase, which contains a variety of mucins adjacent to the glycocalyx of the apical cells of the epithelium. Ocular surface mucins adhere to the glycocalyces of conjunctival and corneal epithelial cells and enhances the wettability of the cornea by serving as an interface between the hydrophobic corneal epithelium and the aqueous tear fluid [1,2]. Therefore, mucin can stabilize the tear film, provide a smooth and refractive surface of high optical quality over the cornea, lubricate the corneal and conjunctival epithelial surfaces during eye blinking, and prevent desiccation of the ocular surface through water retention resulting from heavy O-linked glycosylation. It also serves as a barrier to microbial invasion and shields conjunctival and corneal cells from surface debris and noxious substances [3,4].

Fifteen epithelial mucin genes have been completely or partially sequenced. They are *MUC1*, *MUC2*, *MUC3A*, *MUC3B*, *MUC4*, *MUC5AC*, *MUC5B*, *MUC6*-*MUC9*, *MUC11*-*MUC13*, and *MUC16* [5-10]. According to their physiologic functions, they can be further classified as gel-forming mucins (MUC2, MUC5AC, MUC5B, and MUC6), soluble mucins (MUC7 and MUC9), and membrane-spanning mucins (MUC1, MUC3A, MUC3B, MUC4, MUC12, MUC13, and MUC16). To date, at least five mucins have been found in the ocular surface epithelium. Conjunctival goblet cells secrete the gel-forming mucins, MUC5AC and MUC2, whose expression at the ocular surface is limited to the goblet cells of the conjunctiva [11], whereas the conjunctival and corneal epithelia produce the membrane-spanning mucins, MUC1, MUC4, and MUC16 [11,12].

Goblet cell mucin secretion is tightly regulated since both increases and decreases in mucin in the tear film are associated with ocular surface diseases. Lemp [13] has divided diseases of the mucous layer into diseases of mucous overproduction and deficiency. Diseases of mucous overproduction include giant papillary conjunctivitis, vernal conjunctivitis, atopy, ocular allergy, mucous fishing syndrome, and early dry eye syndromes. Diseases of mucous deficiency include anesthetic cornea (neurotrophic keratitis), herpetic keratitis, familial dysautonomia, alkali burns, radiation burns, Stephens-Johnson syndrome, cicatricial pemphigoid, and late dry eye syndromes.

Patients with vernal keratoconjunctivitis have been shown by impression cytology to have a significant increase in the number of goblet cells when compared to the number of goblet cells in normal control subjects [14]. An increased expression of *Muc5AC* mRNA was also noted in murine and human allergic diseases [15,16]. However, two other studies of goblet cells in a guinea pig model of allergic conjunctivitis demonstrated a decrease in conjunctival goblet cells [17,18]. Thus, the relationship between the number of goblet cells and mucin gene expression in the ocular allergic response is unclear.

Patients with Sjögren syndrome have reduced levels of the gel-forming mucin, MUC5AC, in tears, which correlates with a reduced number of *MUC5AC* transcripts in conjunctival goblet cells and is responsible for decreasing the fluid on wet epithelial surfaces [19,20]. Argüeso et al. [20] showed that some cases of dry eye are secondary to a mucus deficiency leading to decreased tear breakup time. Furthermore, the conjunctival goblet cell density is found to decrease by 86% in keratoconjunctivitis sicca and even more in extreme types of dry eye such as ocular pemphigoid and Stevens–Johnson syndrome [21,22]. However, mucus threads on the ocular surface are often complained by these patients with the status of their goblet cell deficiency.

Past studies have demonstrated that targeted disruption of a specific cell lineage can be a useful method for evaluating its function. In these approaches, a toxin gene driven by a tissue-specific promoter is used to achieve genetic ablation of a particular cell lineage. Targeted expression of γ2-crystallin promoter fused to the diphtheria toxin (DT) A-chain gene (*DT-A*) caused microphthalmia in experimental transgenic mice [23]. Similar targeted expression of the elastase I promoter fused to *DT-A* caused aplasia of the pancreas in transgenic mice [24].

In this report, we used a transgenic approach to selectively ablate conjunctival goblet cells by diphtheria toxin via the expression of a minigene containing the human *MUC5AC* promoter [25] and an attenuated diphtheria toxin A-chain gene [26,27]. This transgenic mouse was used to study the consequences of goblet cell loss on mucin production by examining the mucin changes in the conjunctiva, which might be similar to the mucus-deficient diseases.

## Methods

### Generation of transgenic mouse lines

The 680 bp attenuated diphtheria toxin A cDNA (*Tox176*) was removed from the plasmid, Ktnpr-DTa/BpA (a generous gift from Dr. Winston Kao), by HindIII digestion. The luciferase reporter cDNA in plasmids containing the 4,068 bp (extending from −4.0 kb to +68 bp) human *MUC5AC* promoter in a PGL3 luciferase reporter vector (Promega, Madison,WI) was excised by digestion with NotI and XhoI and was replaced with the attenuated diphtheria toxin A cDNA (*Tox176*). The human *MUC5AC* with attenuated diphtheria toxin A cDNA was separated from vector sequences by agarose gel electrophoresis, purified by glass bead extraction (Geneclean II, BIO 101 Inc., Vista, CA), suspended in TE buffer (10 mM Tris, pH 7.4, 0.2 mM EDTA), sterilized by passage through a 0.22-mm filter (Ultrafree-MC, Millipore, Billerica, MA), and microinjected into the pronuclei of C57BL/6J fertilized mouse oocytes by the Transgenic Core Facility at the Medical College of National Taiwan University (Taipei, Taiwan). Transgenic founders were identified by Southern hybridization of mouse tail DNA digested with HindIII and BamHI using a ^32^P labeled diphtheria toxin A (DTA) probe [25,27-29]. Animal experiments were performed in compliance with the ARVO Statement for the Use of Animals in Ophthalmic and Vision Research. Animals were housed in a room maintained at 23 °C with a fixed 12 h light/dark cycle (lights on from 6 AM to 6 PM) and given free access to Purina chow (Purina, St. Louis, MO) and water. On the morning of the experiment, animals were anesthetized with pentobarbital sodium (5 mg per 100 g bodyweight by intraperitoneal injection).

The transgenic mice were identified by polymerase chain reaction (PCR) of mouse tail DNA with a primer pair chosen from within *Tox176* (5′-AAC TTT TCT TCG TAC CAC GG-3′ and 5′-ACT CAT ACA TCG CAT CTT GG-3′) and another primer pair for the *MUC5AC* promoter (5′-GGA AAC TGG GCT CTA CCC GG-3′ and 5′-CAT TGT GTG GAC GGC GGG GA-3′). Male mice heterozygous for the *MUC5AC*-*Tox176* transgene were bred with female mice heterozygous for the *MUC5AC*-*Tox176* transgene to produce mice of four separate genotypes: those carrying both transgenes, those carrying one of the two, and those carrying neither transgene. The presence of the transgenes was determined by PCR analysis of genomic DNA obtained from tail biopsy and prepared using the QIAmp tissue isolation kit (Qiagen, Valencia, CA). Sex-matched littermate mice between the ages of two and four months were used for all experiments.

### Visualization and quantitation of conjunctival goblet cells

To visualize and quantify the filled conjunctival goblet cells, a modified conjunctival whole mount technique was used [30,31]. The eyes were enucleated, and whole mounts of inferior conjunctiva were dissected and fixed overnight in 4% paraformaldehyde. These tissues were then rinsed three times (10 min per rinse) in phosphate buffer saline (PBS; 75.2 g K_2_HPO_4_, 13.2 g NaH_2_PO_4_·H2O, and 72 g NaCl dissolved in 1 liter of distilled water). The specimens were treated with 3% glacial acetic acid for 3 min. They were then incubated with Alcian blue (AB, pH 2.5; Muto Chemicals, Tokyo, Japan) for 40 min and with 0.5% periodic acid solution (Fisher Scientific, Pittsburgh, PA) for 5 min. The tissues were then flat-mounted on a glass slide in Vectashield mounting medium (Vector Laboratories, Burlingame, CA).

Images were imported into image management software (Photoshop, version 5.0; Adobe, San Jose, CA), and measurement of the entire epithelial area was achieved by tracing the area with the program’s “lasso” tool. The total data area, measured in pixels, is acquired through the “image:histogram” command in the program. Two independent counts are recorded for goblet cells. Goblet cells per unit area of pixels were adjusted to real unit area or cells per square millimeter of real epithelial area based on 28.346 pixels/cm in the software and a calibration factor of 1 mm=4096 pixels at 40X magnification on the confocal microscope. Data were recorded as goblet cells per square millimeter, and the results were analyzed on the computer with ANOVA (StatView software, version 5.0; Abacus Concepts, Berkeley, CA).

### Immunohistochemical staining

A detailed morphologic analysis of hematoxylin-eosin stained, 4 μm thick slides obtained from formalin-fixed, paraffin-embedded tissue blocks was performed. Representative sections were stained with periodic acid–Schiff (PAS), Alcian blue, and mucicarmine. For all mucin detections, antigen retrieval was performed by microwaving rehydrated sections in 10 mM citric acid (pH 6.0) four times for 5 min each with 1 min between each heating. Monoclonal antibody against MUC1, MUC2, MUC4, MUC5AC, anti-phospho-p38-Mitogen-Activated Protein Kinase (p38MAPK), Activating Protein 2 (AP2), anti-phospho-Extracellular Signal-Regulated Kinase (ERK), and cytokeratin-7 (Santa Cruz Biotechnology, Santa Cruz, CA) was applied at a 1:40 dilution at 4 °C overnight. Histochem-Plus kits (Zymed, S. San Francisco, CA) were used to detect bound primary antibody.

### Electron microscopy

Samples were fixed in 2.7% glutaraldehyde and 0.8% paraformaldehyde in 0.1 M cacodylate buffer (pH 7.4) for 2 h at room temperature. They were rinsed in distilled water and post-fixed in 2% osmium tetroxide for 1 h, dehydrated through graded alcohols to 100% ethanol, and embedded in Eponate resin (Polysciences Inc., Warrington, PA). Seventy nanometer sections were cut and stained with 10% uranyl acetate in 50% ethanol and Reynolds lead citrate (microwave method). A Philips Bioscan Techni 10 (Philips, Eindhoven, Netherlands) was used at 80 kV for photography.

### Apoptosis detection by Terminal Deoxynucleotidyl Transferase Biotin-dUTP Nick End Labeling (TUNEL) staining

Four micrometer thick slides were obtained from cold, paraformaldehyde fixed embedded tissue for 6 h and dehydrated in a graded ethanol series (50%, 70%, 85%, 95%, and 100%) followed by 20 min in acetone at −20 °C. The tissue section slides were further permeabilized by incubation in PBS with 0.5% Triton X-100 and 0.1% sodium citrate for 15 min and in a 20 μg/ml proteinase K (Invitrogen, Carlsbad, CA) for 10 min. The samples were refixed with 4% paraformaldehyde before incubation with terminal deoxynucleotidyl transferase (TdT) solution according to the manufacturer’s instructions (in situ cell death detection kit; Hoffmann-La Roche Co. Ltd., Basel, Switzerland).

### Real-time reverse transcription polymerase chain reaction to determine relative *MUC1*, MUC2, *MUC5AC*, and *MUC4* expression

Mouse conjunctivas were snap frozen in liquid nitrogen, and total RNA was isolated by a single-step extraction technique with reagent (TRIzol; Gibco-Life Technologies, Grand Island, NY). The first strand of cDNA was synthesized from 1 µg of DNase-treated RNA with random primers using reverse transcriptase (SuperScript II; Gibco-Life Technologies) followed by RNase H treatment (Invitrogen, Carlsbad, CA). Real-time reverse transcription (RT)–PCR was performed using a sequence detection system (TaqMan PCR GeneAmp 5700; PE Applied Biosystems, Foster City, CA) essentially as previously described [32,33]. To normalize *MUC1*, *MUC2*, *MUC4*, and *MUC5AC* expression relative to cDNA, we used primers and a TaqMan probe corresponding to *β-actin*. The *MUC5AC*-, *MUC4*-, *MUC1*-, *MUC2*-, and *β-actin*-specific primers provided in the public domain by the National Center for Biotechnology (NCBI) [34] are shown in Table 1.

**Table 1:** Primer sequences used for quantitative RT-PCR.

**Gene**	**Primer sequence**
*MUC5AC*	sense 5′-AAAGACACCAGTAGTCACTCAGCAA-3′
	antisense 5′-CTGGGAAGTCAGTGTCAAACCA-3′
*MUC4*	sense 5′-CTCCAAGAAATGTAGTGGCTTTCA-3′
	antisense 5′-CACGGTCTTGGGCTGGAGTA-3′
*MUC1*	sense 5′-TCATCTCAGGACACCAGCAG-3′
	antisense 5′-AGCTGAAGAGGTGCCACTGT-3′
*MUC2*	sense5′-ACGATGCCTACACCAAGGTC-3′
	antisense 5′-GTCCTCCAGTTCTCAGGTCG-3′
*β-Actin*	sense 5′-TCACCCACACTGTGCCCATCTACGA-3′
	antisense 5′-CAGCGGAACCGCTCATTGCCAATGG-3′

For relative quantitation as performed in this study, a comparative threshold (C_T_) method normalizes the amount of target compared with an internal standard control—for example, a suitable housekeeping gene. The relative amount of a target gene in different samples is determined and compared with the amount in the naïve control specimens (calibrator specimen). The internal standard control gene is amplified using rodent *β-actin*. To verify the validity of using *β-actin* as the internal calibration standard, the efficiencies of the *MUC5AC*, *MUC4*, and *β-actin* amplifications were compared and found to be equivalent. The identities of the *MUC5AC* and *MUC4* PCR products were verified by sequencing performed by the DNA Sequencing Core Facility of the National Taiwan University Hospital.

Each PCR reaction contained equivalent amounts of cDNA. Assays were performed in triplicate using a kit according to the manufacturer’s recommendations (TaqMan PCR Reagent Kit; PE Biosystems). Relative quantitation of the amounts of *MUC1*, *MUC5AC*, and *MUC4* mRNAs at 0 h, 6 h, 24 h, and 48 h after final antigen challenge was determined as previously described [32]. The results were analyzed using the unpaired *t*-test.

### In situ hybridization techniques for *MUC4* and *MUC5AC*

In situ hybridization was performed on sections from mouse tissue using DIG-labeled antisense oligonucleotide probes corresponding to the tandem repeat regions of *MUC4* and *MUC5AC*. The sequences used for the DIG-labeled 5′ end of *MUC1* antisense, *MUC2* antisense, *MUC4* antisense, and *MUC5AC* antisense oligonucleotide probes are shown in Table 2. Techniques used are as previously described [35]. Briefly, paraffin sections were deparaffinized and treated with proteinase K. The samples were acetylated and hybridized overnight with 10 pmole DIG-labeled probes. To remove nonspecific binding, slides were subjected to a stringent wash with 0.5X SSC at 65 °C and treated with 20 μg/ml RNase (Sigma, St. Louis, MO) at room temperature for 1 h followed by washing with 0.2X SSC at 65 °C. The hybridization signals were visualized with anti-DIG antibody alkaline phosphatase conjugates using procedures recommended by the manufacturer (Hoffmann-La Roche Co. Ltd.). Finally, the sections were counterstained with 0.5% neutral red and mounted. Images were obtained using an AxioCam digital camera (Carl Zeiss Co., Oberkochen, Germany) on Stemi SV11 Apo (Zeiss) dissection microscope. As a control, in situ hybridization using a sense oligonucleotide probe for *MUC4* and for *MUC5AC* (Table 2) was performed on all tissues.

**Table 2:** Sequences used for the DIG-labeled 5′ end of oligonucleotide probes.

**Gene**	**Sequence**
*MUC5AC*	antisense oligonucleotide, 5′-GGTTGTAGAGATGGTGCTGGTCTTTCCTGTATTGGGTGAGCTGGTTTG-3′
	sense oligonucleotide probe, 5′-CAAACCAGCTCACCCAATACAGGAAAGACCAGCACCATCTCTACAACC-3′
*MUC4*	antisense oligonucleotide, 5′-TGTAGAACCTTGAGTCCTTACTGCTGTTGTGTGTCCTGTG-3′
	sense oligonucleotide probe, 5′-CACAGGACACACAACAGCAGTAAGGACTCAAGGTTCTACA-3′
*MUC1*	antisense oligonucleotide, 5′-ACTGGTAGAGTCTTCAGGAGCTCTGGTGGCTGGAGAAGAGGTGCTACT-3′
*MUC2*	antisense oligonucleotide, 5′-GGAGGGTGTTGGTGTTGGTGTTGAGGGTGTAGGAGTTGAGGGAGTAGA-3′

## Results

### Goblet cell–specific reporter gene expression by human *MU5AC* promoter in transgenic mice

**Figure 1:**
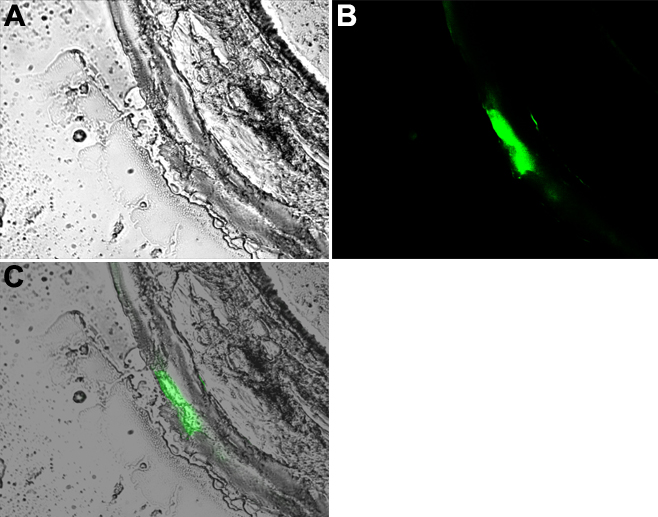
Goblet cell–specific expression of EGFP by human *MUC5AC* promoter in transgenic mice. Transgenic mice were produced by an injection of a construct composed of the 4.0 kb of the 5′ end of human *MUC5AC* promoter that was ligated to EGFP. Tissues were fixed and observed under fluorescent microscopy to locate the expression of EGFP as detailed in Methods. **A**: Differential interference contrast (DIC) picture of transgenic mouse conjunctiva. **B**: Frozen section of conjunctival tissue with EGFP expression in the same section. **C**: Merged picture of **A** and **B**.

A construct comprising 4.0 kb of the human MUC5AC peomoter ligated to 5’ end of the reporter Enhanced Green Fluorescent Protein (EGFP) was used to establish transgenic murine lines. As demonstrated in Figure 1, the reporter EGFP, which has been driven by the human *MUC5AC* promoter, was expressed in conjunctival goblet cells. The same pattern of expression was observed in each of four lines derived from individual founder mice. Of note, EGFP was not detected in other ocular tissues, confirming both the cell- and tissue-specificity of human *MUC5AC* promoter–driven gene expression.

### Characterization of human MUC5AC-DTA transgenic mice

**Figure 2:**
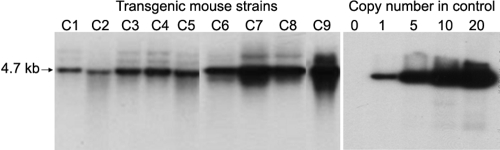
Southern blot analysis to determine the copy number of the human *MUC5AC*-*DTA* transgene in human *MUC5AC*-*DTA*(+/−) transgenic mice. Arrow at the left indicates the 4.7 kb *MUC5AC*-*DTA* transgene construct in the control and the fragments liberated from the genomic DNA of transgenic mice by HindIII digestion. The copy number of the transgene varied from 1 to 10 among the nine founder lines. Transgenic mouse lines, C1, C2, C3, C4, and C5, had approximately one copy of the minigene inserted into the genome of transgenic mouse line. C6, C7, C8, and C9 had more than one copy of the minigene inserted in tandem.

A 4.0 kb human *MUC5AC* promoter was used to create transgenic mice that overexpressed a 680 bp attenuated diphtheria toxin A cDNA (*Tox176*). Nine transgenic founders (F0) were generated and identified by PCR and Southern blot analysis. They were all established as stable transgenic mouse lines by breeding with C57BL/6J mice (Figure 2). The integrity of the transgene DNA and its copy number were determined by Southern blot analysis with HindIII, which makes a single cut at the 345th bp of the minigene. A 4.7 kb genomic DNA fragment was hybridized to the ^32^P-labeled cDNA of diphtheria toxin. The copy number of the transgene varied from 1 to 10 among the nine founder lines. Transgenic mouse lines, C1, C2, C3, C4, and C5, had approximately one copy of the minigene inserted into the genome of transgenic mouse line. C6, C7, C8, and C9 had more than one copy of the minigene inserted in tandem, and overexposure of the Southern blot hybridization did not yield evidence to support multiple insertion sites in the genome. Those mice with multiple copies of the minigene were not used for further analysis because they were infertile. This phenomenon may be a toxic effect to the germ cells due to the ectopically expressed transgene. Those mice with multiple copies of the minigene might have a more toxic effect on the germ cells than those with one copy. The mice with one copy of the minigene were successfully obtained and then bred. However, these mice also lose their fertility gradually after four to five generations due to the toxic effect to germ cells.

**Figure 3:**
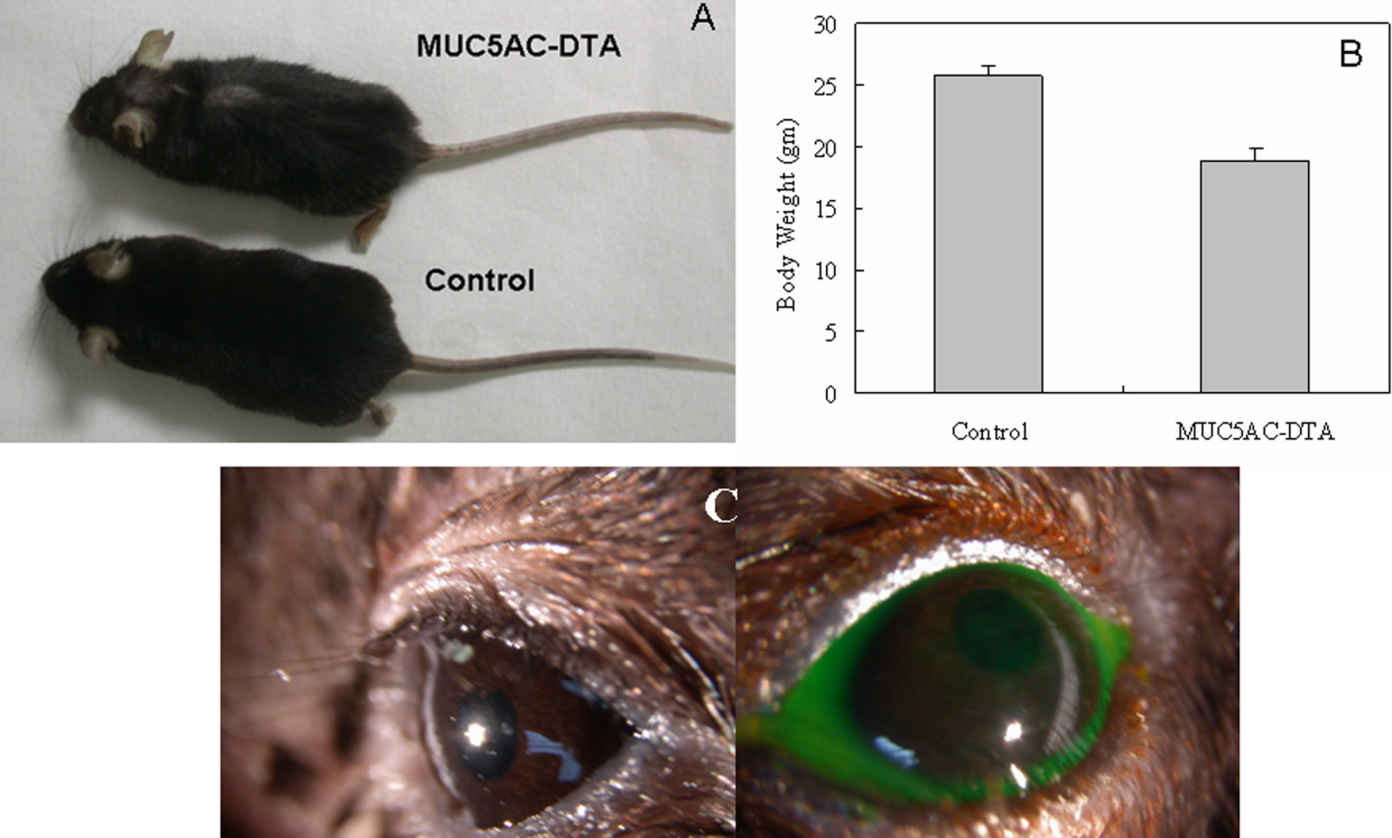
Phenotypes of human *MUC5AC*-*DTA* transgenic mice. **A**: The transgenic mice are smaller than normal mice and have prominent hair loss. **B**: The bodyweights of transgenic mice are less than those of normal mice at the age of three months. **C**: The left image shows the normal external eye of the transgenic mouse and the right image shows the fluorescein staining of the cornea. There is no fluorescein staining in the cornea and the conjunctiva of transgenic mice.

**Figure 4:**
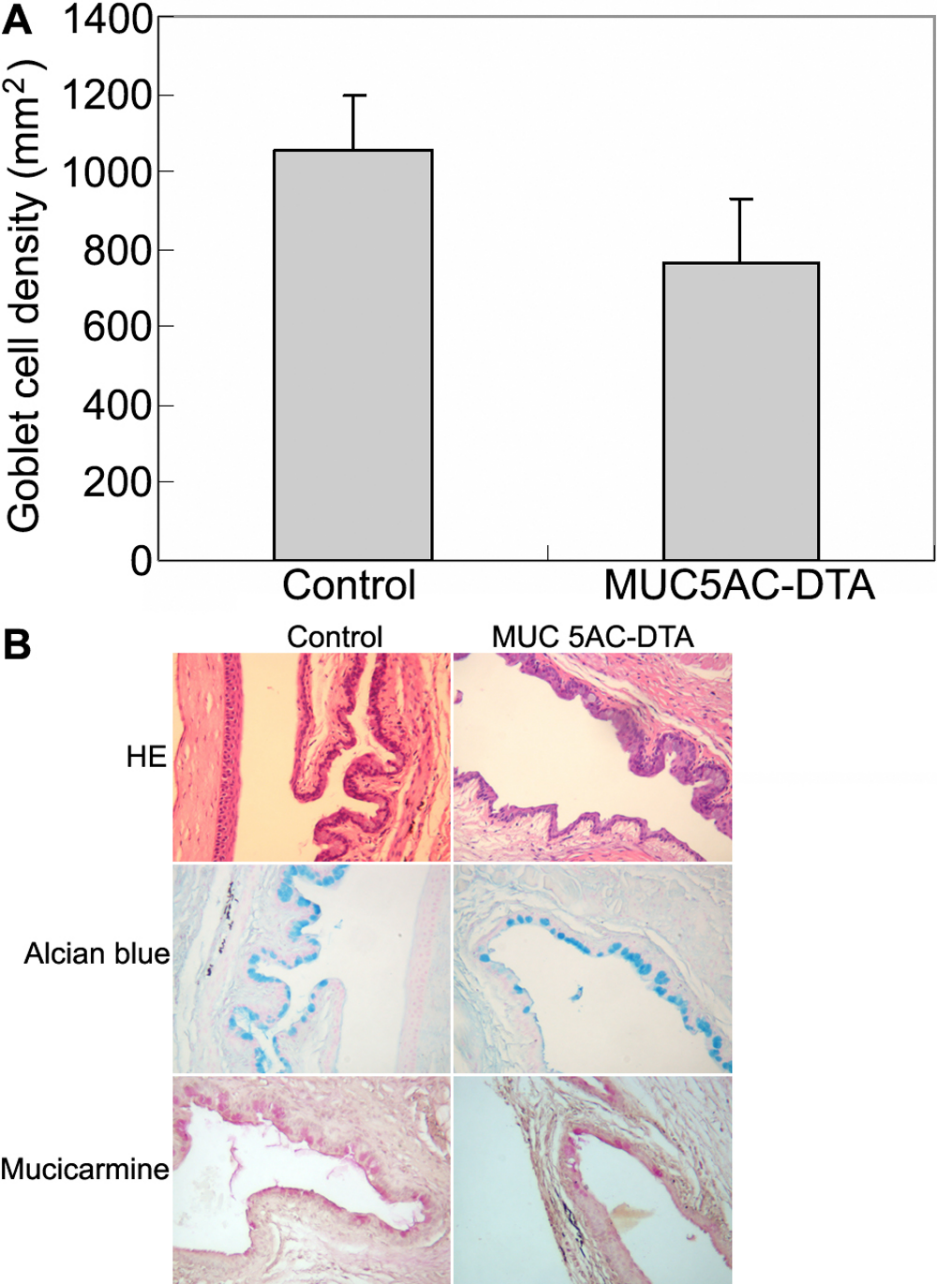
Expression of mucins in conjunctivas of normal mouse and transgenic mouse. **A**: Visualization and quantitation of conjunctival goblet cells are shown. Whole mount Alcian blue staining was used to count the number of goblet cells in the mouse conjunctival epithelial sheets. The density of goblets cells in transgenic mice was less than that of control mice. **B**: The conjunctiva of *MUC5AC*-*DTA* transgenic mouse (left column) and control littermate (right column) were subjected to mucin histological examination (100X). In Alcian blue staining (middle panel) and mucicarmine staining (lower panel), the expression of mucin decreased in transgenic mouse.

The transgenic mice are smaller than normal mice, and hair loss is prominent in transgenic mice (Figure 3A). The bodyweights of transgenic mice are less than those of normal mice at the age of three months (n=5, Figure 3B). However the external eye, which is the cornea and conjunctiva, looks normal, and there is no fluorescein staining after application of fluorescein dye in the conjunctival sac (Figure 3C). To determine the density of goblet cells in conjunctiva, whole mount Alcian blue staining was used to count the number of goblet cells in the mouse conjunctival epithelial sheet. The density of goblets cells in transgenic mice was less than that of control mice (n=5, Figure 4A).

**Figure 5:**
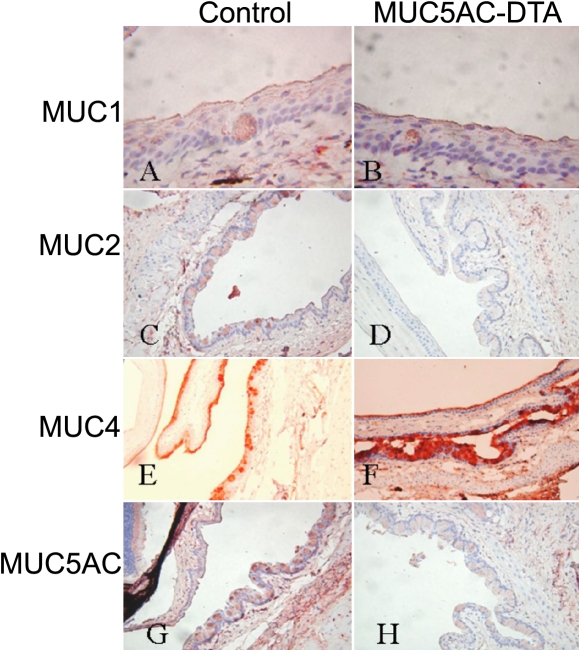
Immunohistochemical staining against different mucins in conjunctivas. In immunohistochemical stainings (100X), both transgenic mice and control mice expressed the normal patterns of Muc1 (**A** and **B**). The expression of MUC5AC (**G**,**H**) and MUC2 (**C**,**D**) decreased in transgenic mice. The intensity of MUC4 immunostaining increased in transgenic mice (**E**,**F**).

To examine the morphological changes in human MUC5AC-DTA transgenic mice, the conjunctiva of transgenic mice and non-transgenic littermates were subjected to histological examination. In Alcian blue and mucicarmine stainings, the expression of mucin decreased in transgenic mice (Figure 4B). In immunohistochemical stainings, both transgenic mice and control mice expressed normal patterns of MUC1. However, the expression of MUC5AC and MUC2 decreased in transgenic mice. The intensity of immunostaining of MUC4 increased in transgenic mice (Figure 5).

**Figure 6:**
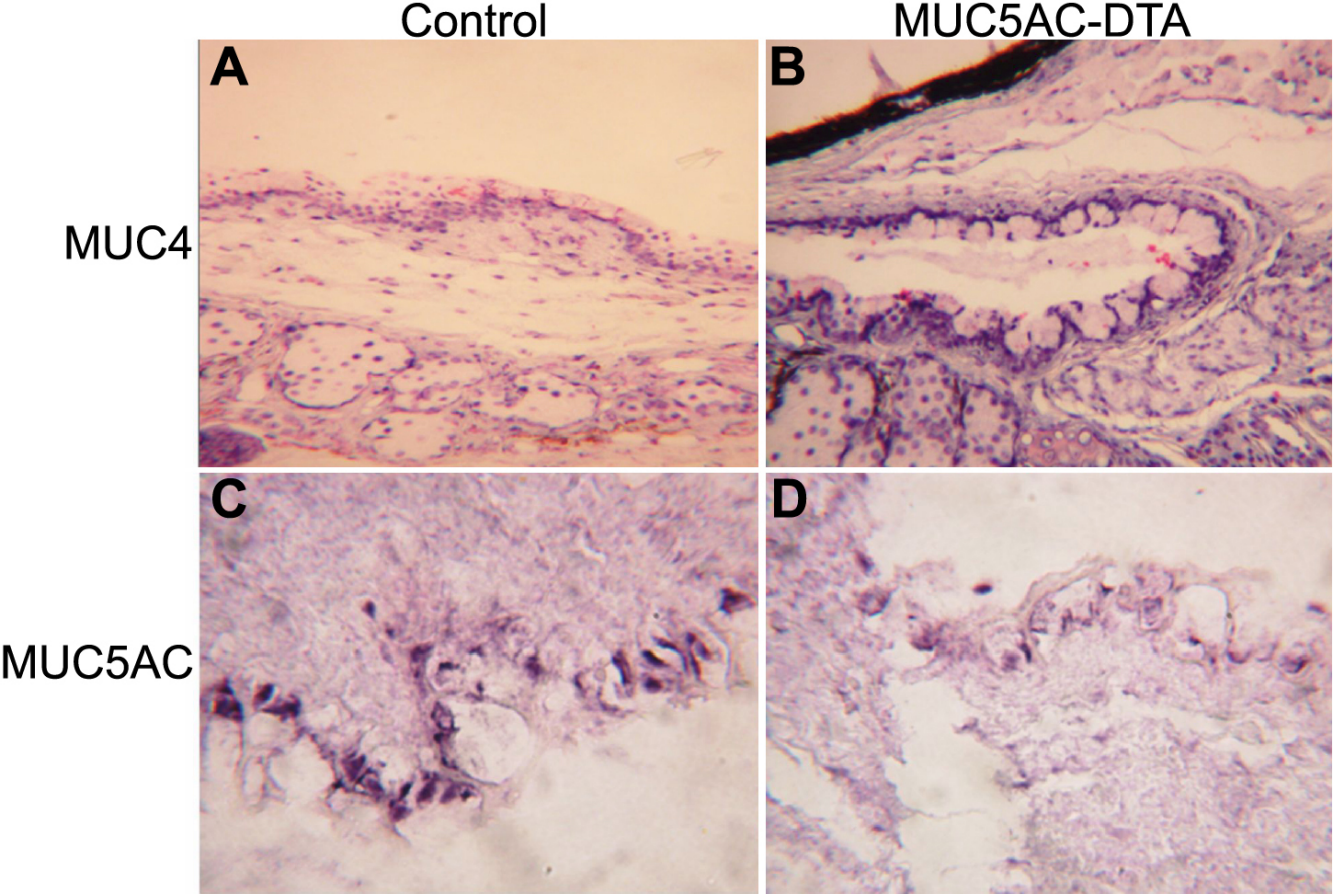
In situ hybridization of *MUC4* and *MUC5AC* mRNA. *MUC5AC* mRNA decreased (**D**) and *MUC4* mRNA (**B**) increased expression in the conjunctiva of transgenic mice compared with those of their control littermates (**A** and **C**). Magnification, 200X.

**Figure 7:**
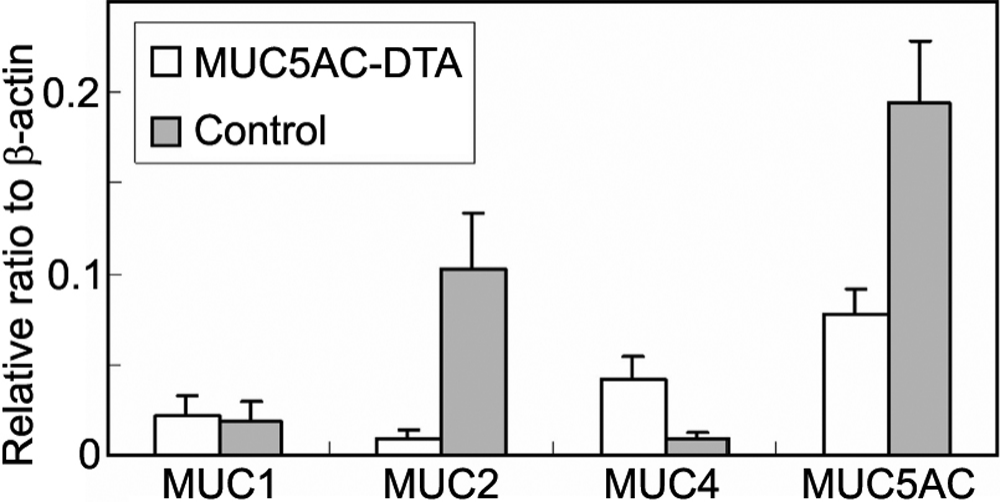
Expression of different mucin mRNA in conjunctivas. Quantitative RT–PCR was used to measure mucin mRNA. The expression of *MUC2* mRNA and *MUC5AC* mRNA decreased while the expression of *MUC4* increased in the conjunctivas of transgenic mice compared with those of their control littermates.

In situ hybridization showed that there was also decreased expression of *MUC5AC* mRNA and increased expression of *MUC4* mRNA (Figure 6). We also used quantitative RT–PCR to measure the mRNA of mucin expression in transgenic mice. We found that the expression of *MUC2* and *MUC5AC* decreased in transgenic mice. However, *MUC4* expression increased in transgenic mice (Figure 7).

**Figure 8:**
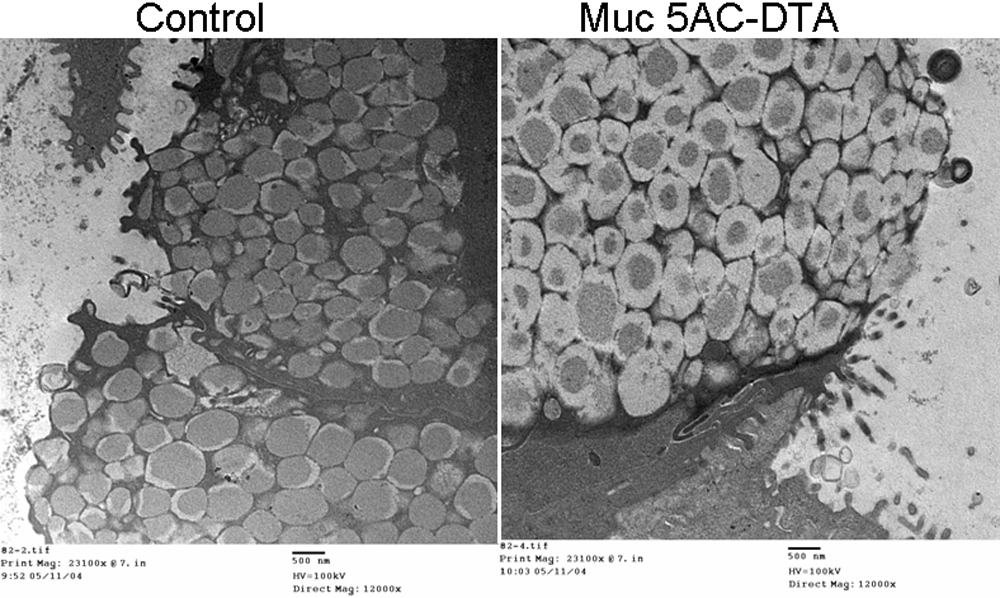
Electron microscopic findings of secretory granule in conjunctivas. Electron-dense materials (secretory mucins) in the secretary granules decreased in transgenic mice (right) compared with those of their control littermates (left). Magnification, 12,000X.

**Figure 9:**
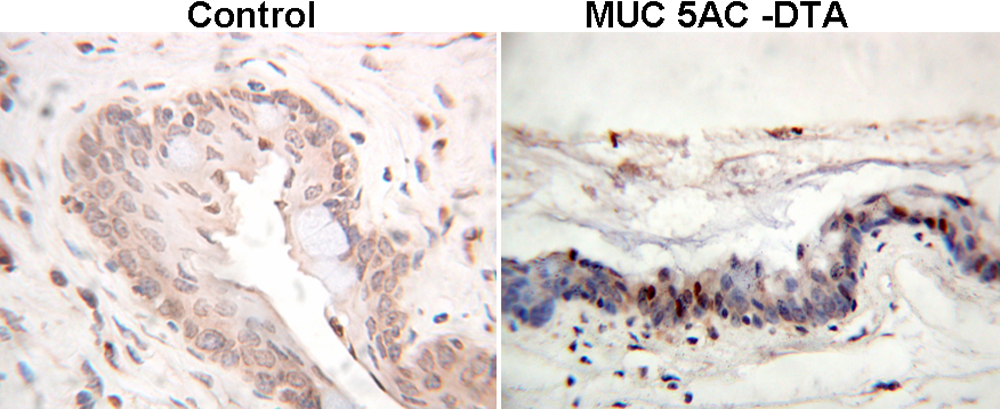
Apoptotic cells in conjunctivas. TUNEL staining was used to study apoptotic cells in conjunctival epithelium (200X). There were significantly more TUNEL-positive cells in the MUC5AC-DTA mice (right) compared with those of their control littermates (left).

An electron microscopy study revealed that electron dense materials in the secretary granules, which contain the secretory mucins in goblet cells, decreased in transgenic mice (Figure 8). Finally, TUNEL staining was used to study apoptotic cells in conjunctival epithelium. We found that TUNEL-positive cells can be found in the conjunctival epithelium, especially in goblet cells (Figure 9).

**Figure 10:**
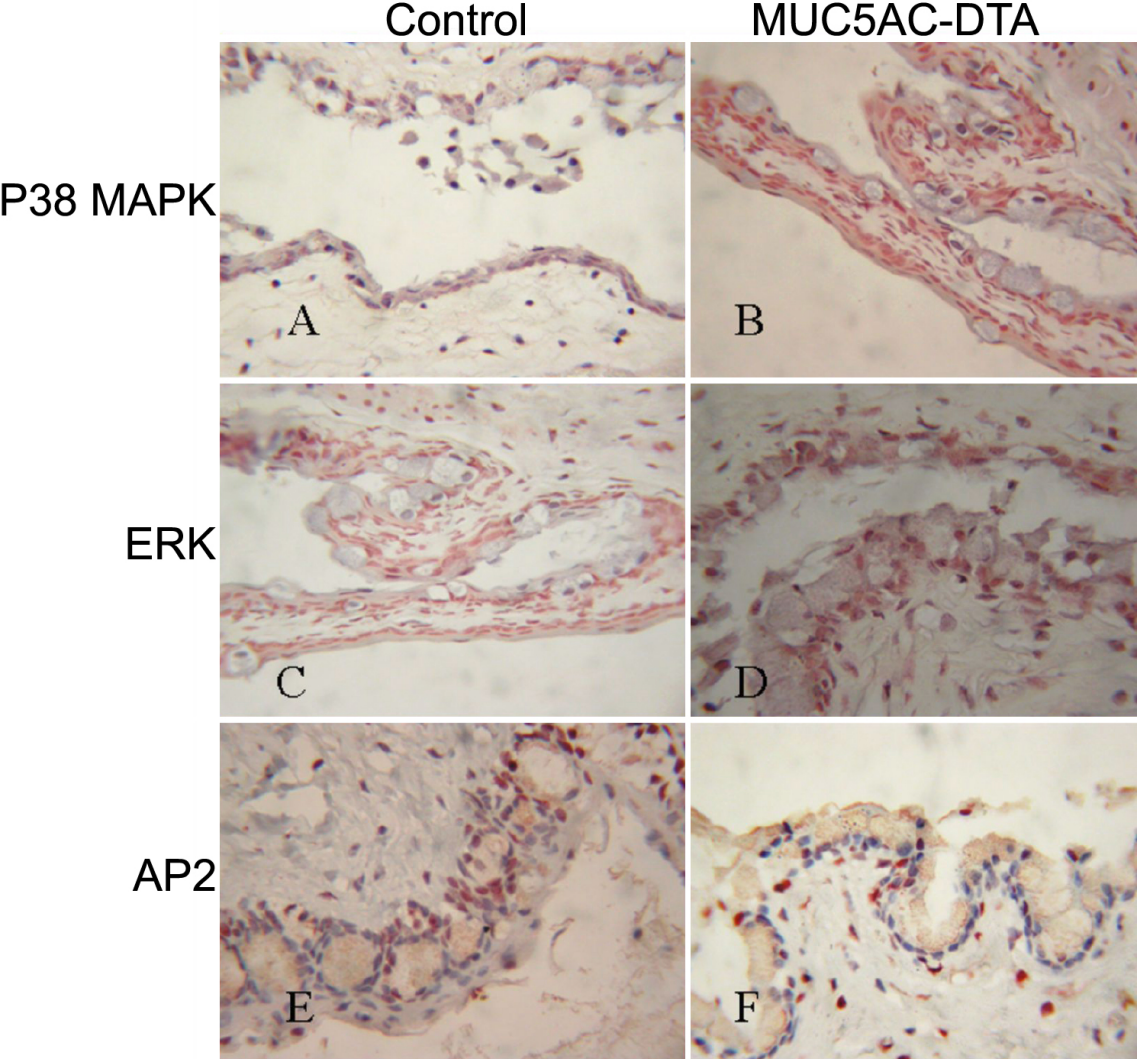
Possible mechanisms of the phenotypes of MUC5AC-DTA. In immunohistochemical stainings (100X), the expression of anit-phospho-p38MAPK was detected in conjunctival goblet cells and conjunctival epithelial cells of transgenic mice but not in control mice (**A**,**B**). Both transgenic mice and control mice expressed the similar patterns of anti-phospho-ERK (**C**,**D**) and AP2 (**E**,**F**). The compensatory overexpression of MUC4 in MUC5AC-DTA mice is possibly mediated through the expression of p38MAPK.

A positive staining of ant-phosphop38MAPK could be detected in the conjunctival goblet cells and conjunctival epithelial cells of transgenic mice but not in the control mice (Figure 10A,B). Similar staining patterns could be observed for anti-phospho-ERK and AP2 in conjunctivas between transgenic mice and control mice (Figure 10C-F).

## Discussion

In the current study, we used a transgenic mouse model to mimic goblet cell deficient conjunctivitis as mentioned above. Our results confirmed the hypothesis that MUC4 mucin can compensate for the decrease of secreted MUC5AC mucin in diseases of goblet cell deficiency as reported by Dogru et al. [36]. Overexpression of these mucins may also explain the excessive mucus discharge observed in eyes with allergy or early dry eye diseases. In allergic diseases, it is possible that the initial response of the atopic ocular surface epithelium to inflammation in regard to mucin secretion may be a downregulation of epithelial mucins relative to healthy control eyes [36]. The goblet cell densities and mRNA expressions of *MUC5AC* showed a dramatic reduction in atopic patients with a severe epithelial disease, especially in patients with corneal ulcers, relative to patients without any epithelial disease and healthy control subjects [36]. They suggested that based on the analysis of *MUC1*, *MUC2*, and *MUC4* mRNAs, the ocular surface epithelial cells might be secreting other mucins to compensate for the decrease of MUC5AC and to protect the ocular surface [37]. Our results also demonstrated the same trend of mucin compensation described by Dogru et al. [36].

There are notable differences between the Dogru et al. [36] study and ours. First, we used an animal model that lacks atopic eye diseases as well as a normal morphology of the cornea and conjunctiva. Thus, there will be some differences in mucin expression between species. Second, the expression of *MUC1*, *MUC2*, and *MUC4* are upregulated in the Dogru et al. [36] study. In our study, only *MUC4* was upregulated. The differential expression of mucin genes in different ocular diseases was also noted in the previous report. For example, both the membrane-spanning mucin, asialoglycoprotein (ASGP), and the gel-forming mucin, rMuc5AC, were downregulated by vitamin A deficiency in rat ocular surface epithelium [38]. Danjo et al. [39] reported that MUC4 may be the source of mucin that compensates for loss of MUC1 in some strains of *MUC1* null mice, which do not have ocular surface anomalies. The mRNA level of *MUC4* in the ocular surface tissue of the *MUC1* null mice was not upregulated when compared with that of control mice through real-time PCR, an observation that has also been reported by another paper that used slot-blot analysis of mRNA of the mammary gland, salivary gland, lung, stomach, and colon of *MUC1* null mice [40]. The possible cause of these differences is that there is a high level of expression of the membrane-spanning mucin, *MUC4*, in the conjunctiva and cornea of rats and mice [38,41]. In humans, the level of expression of *MUC4* in the central cornea is much lower [11,42]. It is also possible that these differences reflect the relatively high concentration and importance of MUC4 on rodent eyes relative to that of MUC1, which has a negligible effect on the rodent ocular surface. Therefore, in our study, *MUC4* is upregulated instead of *MUC1*.

Another possible way in which mucin alteration may be involved in ocular surface diseases relates to changes in mucin glycosylation in our animal model as previously reported [43]. The expression pattern of members of the GalNAc-transferase family, which are the enzymes responsible for initiation of mucin O-glycosylation, is altered during the keratinization process that leads to severe dry eye in ocular cicatricial pemphigoid patients [43]. In the early stages of the disease, GalNAc-T2 and -T6 isoenzymes, normally not expressed in the apical cells of the conjunctival epithelium, are expressed in these cells. There is either a compensatory upregulation of GalNAc-transferases that results in an increase in the density of O-glycan residues, an alteration in O-glycan structure, and/or the glycosylation of additional proteins synthesized by the cell. As the disease progresses and the conjunctival epithelium becomes keratinized, GalNAc-T6 and GalNAc-T2 isoenzymes in the apical stratified epithelia disappear, and the levels of GalNAc-T1, -T3, and -T4 are dramatically reduced. It is possible that the upregulation of membrane-spanning *MUC4* seen in our data might be a compensatory mechanism like that of GalNAc-transferases.

Muc4 was known to interact with erythroblastic leukemia oncogene homolog 2 (erbB2) to enhance tumorigenesis. This interaction was associated with the hyperphosphorylation of Mitogen-Activated Protein Kinase (MAPK) with the overexpression of cyclooxygenase-2 [44]. Our study also confirmed that upregulation of *MUC4* might be through the hyperphosphorylation of MAPK in the transgenic mice. In contrast, other signal transduction pathways such as ERK and AP2 were not involved in the upregulation of the *MUC4* expression.

There have been few studies using transgenic animals to study the function of goblet cells and the expression of mucin. Itoh et al. [45] generated transgenic mice expressing attenuated *DT* gene, which was driven by the intestinal trefoil factor (*ITF*) promoter to assess the functional role of goblet cells in the intestine. The transgenic mice showed partial ablation of the goblet cells, making possible functional assessment of diminished goblet cell populations. Paradoxically, these mice showed marked protection against exogenous toxic agents such as dextran sodium sulfate (DSS) and acetic acid because ITF expression and goblet cell numbers are increased in *mITF/DT-A* mice. They concluded that mucosal protection may be determined more by adequate ITF expression than by goblet cells themselves. In our study, we found that the number of goblet cells decreased with partial ablation by the human *MUC5AC* promoter with *DT-A*. However, as in the results from Itoh et al. [45], we observed compensatory mechanisms for defense against external stimuli in our mice. The transgene appeared to be more highly expressed within the conjunctival goblet cells and was not observed in other cell types. Further, overall tissue specificity was preserved and aberrant expression was not present in tissues that do not normally express goblet cells. These findings suggest that the 4.06 kb of the 5′ flanking sequence, which was used to drive *DT-A* expression, contains sufficient regulatory information to confer cell- and tissue-specificity but may not contain other enhancer elements necessary for expression.

In conclusion, we found that the membranous mucin, *MUC4*, can compensate for the deficiency of the secretory mucin, *MUC5AC*, in goblet cell deficient mice. This compensation may explain why the symptoms of mucus threads can be found in some goblet deficiency diseases, and it may also provide an alternative defensive mechanism in goblet cell deficiency.
